# The evolving landscape in advanced penile cancer

**DOI:** 10.4155/fso.15.7

**Published:** 2015-11-01

**Authors:** Carlo Buonerba, Giuseppe Di Lorenzo, Giuseppe Calderoni, Matteo Ferro, Francesco Perri, Lucia Lombardi, Raffaele Ardito, Piera Federico, Sabino De Placido, Michele Aieta

**Affiliations:** 1 Division of Medical Oncology, Centro di Riferimento Oncologico di Basilicata, I.R.C.C.S, Via Padre Pio 1, 85028 Rionero in Vulture, PZ, Italy; 2 Medical Oncology Unit, Department of Clinical Medicine, Federico II University, Naples, Italy; 3 Divisione di Urologia, Istituto Europeo di Oncologia, Milano, Italy; 4 Medical Oncology Department POC SS Annunziata Taranto, Italy; 5 IOS SrL e Coleman SPA, Naples, Italy

**Keywords:** chemotherapy, immunotherapy, penile cancer, targeted therapy

Penile carcinoma is a rare malignancy with incidence rates which vary in the range of 1–10 cases per 100,000 men according to ethnicity, cultural background, geographic area and social habits [1]. Keratinizing squamous cell carcinomas, similar to carcinomas of nongenital skin, and verrucous carcinoma are the most common histologic variants, while basaloid and warty carcinomas are less prevalent [1]. Prognosis of penile cancer is mainly dependent on stage, with patients with noninvasive disease showing a 5-year cause-specific survival rate approximating 100% [1]. In published series, cancer-specific survival of patients with invasive tumors has ranged from 75 to 93% in patients with cN0 disease, 40 to 70% in those with cN1 disease, 33 to 50% in those with cN2 disease and 20 to 34% in those with cN3 disease [2]. The rarity of penile cancer poses a great challenge for researchers, and improvements in prognosis have been mostly seen in patients with localized cancer. In one cohort study involving 1000 men treated over the course of six decades, the 5-year cancer-specific survival of patients with clinically negative lymphnode improved by 9% in the last 20 years, increasing from 82% of the period from 1956 to 1993 to 91% of the period from 1994 to 2012. This result paralleled the introduction of sentinal node biopsy, and was maintained after adjustment for grade and T stage [3]. While in patients with N0/1 disease surgery is the mainstay of treatment, N2/3 penile carcinoma requires a multidisciplinary approach involving surgery, radiation therapy and chemotherapy, as recurrence has been observed in up to 90% of cases, and it is especially frequent in patients with extranodal extension and involvement of pelvic lymphnodes [2]. In a Phase II trial by Pagliaro *et al*. [4] conducted in 30 men receiving neoadjuvant chemotherapy based on paclitaxel, ifosfamide and cisplatin, 15 patients (50%) had an objective response and 22 (73.3%) subsequently underwent surgery. Of note, three patients (10%) had no remaining tumor on histopathology. Chemotherapy was very well tolerated, with grade 3–4 side effects, including anemia, neutropenia, febrile neutropenia and peripheral neuropathy, occurring each in less than 5% of patients. Surgery, which included inguinal and pelvic lymphadenectomy, was also well tolerated, with perioperative side effects such as noninfectious wound separation, skin breakdown, hemorrhage, skin infection, lower extremity edema and soft tissue necrosis, each occurring in less than 10% of patients. The estimated median time to progression in 20 patients who died during the follow-up was 8.1 months (95% CI: 5.4–50+), with an overall survival of 17.1 months (95% CI:, 10.3–60), while the median duration of follow-up for the 10 surviving patients was 34 months (range, 14–59 months). These findings appear encouraging if compared with existing data [1,2]. As the greatest majority of patients with relapsing disease show inguinal/pelvic recurrence [1], adjuvant radiation therapy may further improve these results. One retrospective study showed that regional failure rates after inguinal lymphnode dissection in 14 men with pathological inguinal lymphnode metastasis after lymphadenectomy were 11% (1 of 9) versus 60% (3 of 5) in patients treated with versus without adjuvant radiation therapy [5]. As shown in Table 1, chemotherapy, biological therapy and immunotherapy are currently being investigated in several ongoing trials. A large prospective trial with a Bayesian design conducted by Nicholson *et al*. is planning to allocate approximately 400 patients to either neoadjuvant chemotherapy or neoadjuvant chemoradiotherapy plus inguinal lymphnode dissection versus inguinal lymphnode dissection only. After inguinal lymphadenectomy, high-risk patients are randomized to pelvic lymphnode dissection versus observation. If this trial accomplishes to reach its target accrual population, it can definitively establish the exact management of locally advanced penile cancer. The need for further therapeutic option is compelling in patients with metastatic disease. Platinum- and taxane-based regimens yielded an overall survival <12 months in the first-line setting [6] and <6 months in the second-line setting [7], respectively. One attractive approach is to combinechemotherapy with biological drugs. In the ongoing study by Boyle *et al*., penile cancer patients are randomized to receive cisplatin–paclitaxel–ifosfamide with or without the addition of the anti-EGFR monoclonal antibody cetuximab. Both EGFR and Ras play an important role in penile cancer, as shown by EGFR overexpression and lack of tumor-suppressor RAS-association domain family 1A protein (RASSF1A) expression in the majority of patients [8]. Although complete responses have been reported with cetuximab, an analysis of 28 patients receiving cetuximab in retrospective series showed a modest PFS of 3 months, and the synergistic effect of cetuximab plus chemotherapy remains to be defined [8]. Of note, other members of the EGFR family may be a suitable biological target in penile cancer, such as HER3 and HER4 [9]. Dacomitinib, which inhibits EGFR, HER2 and HER4, has the potential to be active in penile cancer and is currently being investigated in a prospective trial [10]. In a Phase II study in 48 head-and-neck cancer patients who had progressed on platinum-based chemotherapy, 20.8% of patients had partial responses and 65% of patients had stable diseases, with a median progression-free survival of 3.9 months and an overall survival of 6.6 months [10]. These results are encouraging in view of the known similarities in histology and biology of penile and head-and-neck squamous cell carcinoma. Another biological agent which is currently being investigated in penile carcinoma is pazopanib. A Phase I trial found that pazopanib plus paclitaxel can be safely combined at the usual doses employed when these agents are used separately [11] in view of their different toxicity profile and the absence of any significant pharmacokinetic interaction. Both pazopanib and weekly paclitaxel exert a strong antiangiogenetic activity. Paclitaxel is active in penile cancer [7], while pazopanib inhibits a number of tyrosine kinases, including the VEGF receptor. The VEGF receptor is activated by the VEGF–A ligand which has been found to be overexpressed in approximately 50% of penile cancer cases [12]. Another chemotherapy agent with antiangiogeneic properties, vinflunin, is being investigated in small ongoing trial. Vinflunine is a third-generation bifluorinated semisynthetic vinca alkaloid approved in bladder cancer, and it exerts its antineoplastic activity by acting against tubulin and microtubules and disrupting newly formed blood vessels, with an excellent safety profile [13]. Results of the small ongoing trial by Pickering *et al*. in penile cancer are awaited in 2018. Active immunotherapy holds a great promise in solid tumors [14]. As high-risk Human Papilloma Virus (HPV) is known to be implicated in penile cancer, currently available preventive vaccine directed against HPV 16 and 18 may help reduce incidence of penile carcinoma in selected high-risk populations [15]. Whether active immunotherapy may be effective in HPV-induced cancers (including penile carcinoma) is unknown. In this regard, it must be noted that there is evidence that in HPV-associated cancers presence of tumor infiltrating lymphocytes is associated to a better outcome [16]. Furthermore, presence of HPV isa favorable prognostic factor in penile cancer [1], which may be related to HPV-directed immune response, and partial loss of HLA-A is an independent predictor of poor survival in penile cancer, which underlines the role of the immune response [17]. Two active immunotherapy studies are ongoing in HPV-induced cancers, including penile carcinoma. In both studies, a nonmyeloablative lymphocyte-depleting preparative regimen of cyclophosphamide and fludarabine is administered prior to immunotherapy. One study is evaluating the use of patient derived *in vitro*-expanded tumor infiltrating lymphocytes, which have proven to provide durable responses in patients with melanoma [18]. The other study is investigating the use of T cells genetically engineered with a TCR targeting HPV-16 E6 (E6 TCR).

In conclusion, current management of advanced penile cancer is unsatisfactory, and the prognosis is dismal. Therapeutic advancements are made more difficult by the rarity of the disease. Early recognition of penile cancer is essential, as prognosis worsens dramatically in patients with advanced disease. Similarly to cervical cancer, vaccination may play a role in selected high-risk populations, although further studies are required to assess the cost effectiveness of such an approach. A number of trials are exploring novel therapeutic options, although the majority of them are unlikely to provide definitive evidence of therapy efficacy. Adequately controlled randomized studies conducted in an international setting are required in order to obtain regulatory approval of the high-cost biological agents under investigation.

**Table 1.** **Ongoing interventional trials in penile carcinoma.**

**Principal investigator**	**ClinicalTrials.gov Identifier**	**Main inclusion criteria**	**Sample size**	**Phase**	**Intervention**	**Primary end point**	**Ref.**
Boyle	NCT02014831	Advanced penile carcinoma (N3 or M1)	42	IIb	Cisplatin, paclitaxel and ifosfamide with or without cetuximab	Radiological response rate	[19]
Necchi	NCT01728233	Treatment-naive advanced penile carcinoma (N2/3 or M1)	37	IIa	Dacomitinib	Radiological response rate	[20]
Climent	NCT02279576	Progressive penile carcinoma after treatment with cisplatin or carboplatin-based chemotherapy	32	IIa	Pazopanib plus paclitaxel	Radiological response rate	[21]
Nicholson	NCT02305654	Any T any N M0 treatment-naive penile carcinoma	400	III	ILND vs. neo CT followed by ILND vs. neo CRT followed by ILND. After ILND, patients at high risk are randomized to PLND vs. observation	Overall survival	[22]
Pickering	NCT02057913	M1; M0, any T, N3; M0, any T, N2; M0, T4 any N.	22	IIa	Vinflunine	Radiological response rate	[23]
Williams	NCT01585428	Prior platinum-based therapy HPV+ disease	73 (including other HPV-induced tumors)	II	Fludarabine, cyclophosphamide, followed by IL-2 and TIL	Radiological response rate	[24]
Williams	NCT02280811	Prior standard first-line therapy HPV+ disease	61 (including other HPV-induced tumors)	II	Fludarabine, cyclophosphamide, followed by IL-2 and T-cell targeting HPV-16 E6	Radiological response rate	[25]

CT: Computed tomography; ILND: Inguinal lymph node dissection; PLND: Pelvic lymph node dissection; TIL: Tumor-infiltrating lymphocyte.
